# Clearance of *Strongyloides stercoralis* infection using ivermectin improves kidney function and modulates untargeted urinary metabolomic profile in patients with early-stage chronic kidney disease

**DOI:** 10.7717/peerj.21832

**Published:** 2026-10-29

**Authors:** Keerapach Tunbenjasiri, Suppakrit Kongsintaweesuk, Kornpatchara Chaiyachate, Aye Myat Mon, Thatsanapong Pongking, Apinya Jusakul, Sirirat Anutrakulchai, Apisit Chaidee, Porntip Pinlaor, Nuttanan Hongsrichan, Ubon Cha’on, David Blair, Somchai Pinlaor

**Affiliations:** 1 Biomedical Sciences Program, Graduate School, Khon Kaen University, Khon Kaen, Thailand; 2 Chronic Kidney Disease Prevention in Northeastern Thailand, Khon Kaen University, Khon Kaen, Thailand; 3 Faculty of Associated Medical Sciences, Khon Kaen University, Khon Kaen, Thailand; 4 Department of Parasitology, Faculty of Medicine, Khon Kaen University, Khon Kaen, Thailand; 5 Centre for Research and Development of Medical Diagnostic Laboratories, Faculty of Associated Medical Sciences, Khon Kaen University, Khon Kaen, Thailand; 6 Department of Medicine, Faculty of Medicine, Khon Kaen University, Khon Kaen, Thailand; 7 Department of Biochemistry, Faculty of Medicine, Khon Kaen University, Khon Kaen, Thailand; 8 College of Science and Engineering, James Cook University of North Queensland, Townsville, Australia

**Keywords:** Strongyloidiasis, Kidney disease, Early stage, Metabolic profiling, Helminth treatment

## Abstract

**Introduction:**

*Strongyloides stercoralis* exacerbates the early stages of chronic kidney disease (CKD) through the disruption of metabolic processes. However, the metabolic consequences of infection and of its resolution after treatment remain poorly characterized. This study explores urinary metabolic changes in patients with early-stage CKD following antiparasitic therapy.

**Methods:**

In an exploratory before-and-after longitudinal study design, fifteen patients presenting with concurrent early-stage CKD (stages 1–3) and confirmed strongyloidiasis were monitored at baseline and 17 months following ivermectin anthelmintic treatment. Serum and urine clinical biochemistry assessments were paired with high-resolution untargeted liquid chromatography-quadrupole time-of-flight mass spectrometry (LC/Q-TOF MS) urinary metabolomic profiling executed in both negative and positive ionization modes.

**Results:**

Global multivariate analyses did not demonstrate statistically significant separation between pre- and post-treatment urinary metabolomic profiles. However, univariate analyses identified 91 significantly altered urinary mass features following treatment. Among these, thirteen metabolites (twelve decreased and one increased) were identified as potential markers associated with parasite clearance in patients with CKD. Changes in fasting blood sugar, serum creatinine, and estimated glomerular filtration rate (eGFR) were also observed. Notably, urinary 1-O-galloyl-6-O-cinnamoylglucose was negatively correlated with urine creatinine, while sedoheptulose-7-phosphate levels was negatively correlated with serum creatinine. Additionally, urinary ascorbic acid levels increased following treatment, suggesting their potential relevance to kidney function. Pathway analysis indicated disturbances in amino acid and carbohydrate metabolism, particularly glycine/serine metabolism, tyrosine metabolism, and glycolysis, that partially normalized post-treatment.

**Conclusion:**

These findings suggest that clearance of *S. stercoralis* infection was associated with changes in urinary metabolomic profiles in patients with CKD. Kidney function and urinary metabolomic profiles were observed in patients with early-stage CKD and *S. stercoralis* infection. Further investigations of kidney outcomes following *S. stercoralis* clearance and of the potential value of strongyloidiasis screening in patients with CKD are warranted.

## Introduction

Strongyloidiasis is a neglected tropical parasitic disease caused by *Strongyloides stercoralis*, a soil-transmitted nematode (Buonfrate et al., 2020). It remains endemic across many tropical and subtropical regions, particularly in agricultural areas. Strongyloidiasis imposes a significant global health burden, potentially affecting up to 613.9 million individuals worldwide (Yang et al., 2025). The disease is widespread across Southeast Asia (Laoraksawong et al., 2018), including Thailand, where the prevalence is approximately 11.3% (Chan et al., 2023). In rural communities of northeastern Thailand, the overall prevalence of *S. stercoralis* infection is as high as 23.0% (Laoraksawong et al., 2018). While often asymptomatic in immunocompetent individuals, *S. stercoralis* infection can lead to severe complications in immunocompromised populations, including patients with chronic kidney disease (CKD) (Qu et al., 2016). Given the increasing recognition of interactions between parasitic infections and chronic non-communicable diseases (Wu et al., 2022), understanding the potential impact of *S. stercoralis* infection on CKD has become an important area of investigation.

CKD is a progressive condition that becomes irreversible once it advances to late stages (stages 4–5) (Romagnani et al., 2025). As a leading contributor to both morbidity and mortality, CKD remains a critical global public health challenge (Francis et al., 2024). Worldwide, more than 850 million people are affected by kidney disease in various forms, with the burden disproportionately high in low- and lower-middle-income countries (Jager et al., 2019). In Thailand, the prevalence of CKD is 17.5%, with the highest rates observed in Bangkok (23.9%), followed by the northeastern (22.2%) and northern (20.4%) regions (Cha’on et al., 2022; Ingsathit et al., 2010). Early detection and intervention are critical to slowing CKD progression, particularly during the early stages (stages 1–3), when symptoms are minimal but pathological changes are already underway (Kushner et al., 2024). Accurate renal function assessment remains central to diagnosis and monitoring. In clinical practice, estimated glomerular filtration rate (eGFR), calculated from serum creatinine or cystatin C, is the most widely used indicator. Creatinine-based eGFR has limited sensitivity in detecting early renal impairment and requires invasive blood sampling (Maringhini & Zoccali, 2024), which may delay diagnosis and timely interventions. Recent studies have suggested an association between *S. stercoralis* infection and CKD *via* alteration of the gut microbiome (Hai et al., 2022; Tran et al., 2023b). However, the direct impact of *S. stercoralis* infection on CKD pathogenesis remains poorly understood. Therefore, identifying sensitive biomarkers capable of capturing infection-related metabolic changes may improve our understanding of disease mechanisms and facilitate earlier detection of kidney dysfunction. A novel aspect of this study is the evaluation of urinary metabolomic changes in patients with CKD before and after clearance of *S. stercoralis* infection.

High-throughput analytical platforms have transformed the investigation of complex diseases, enabling earlier diagnosis, improved prognostic evaluation, and more precise treatment strategies (Afrifa-Yamoah et al., 2025). Among these, metabolomics, a powerful technology for comprehensive profiling of small-molecule metabolites, offers a high-resolution view of systemic biochemical states (Lin et al., 2024). It has become an essential tool for identifying metabolic biomarkers, mapping biochemical pathways, and monitoring disease progression, thereby offering valuable insights into disease mechanisms and potential therapeutic targets (Qiu et al., 2023). Recent studies have demonstrated the utility of metabolomics for kidney disease characterization and biomarker discovery, with metabolic fingerprinting further enabling the identification of disease-associated metabolic pathways in renal disorders (Wang et al., 2024b; Xu et al., 2024). Of the various biological samples used in high-throughput studies, urine is particularly valuable due to its non-invasive collection, making it especially suitable for large-scale clinical and epidemiological research.

In this study, we conducted untargeted urinary metabolomics in patients with CKD and concurrent confirmed *S. stercoralis* infection, both before and after successful antiparasitic therapy. We aimed to characterize infection-associated metabolic alterations due to CKD and to assess the impact of these metabolites on kidney function parameters following clearance of *S. stercoralis*, with a particular focus on early-stage patients. This approach provides a novel framework for investigating the relationship between *S. stercoralis* infection, urinary metabolomic profiles, and kidney function in patients with early-stage CKD.

## Materials and Methods

### Ethics statement

All procedures involving human subjects were conducted in accordance with the ethical principles for medical research outlined in the Declaration of Helsinki. The study protocol was reviewed and approved by the Khon Kaen University Ethics Committee for Human Research (Approval Nos. HE661131 and HE671722). Written informed consent was obtained from all participants prior to study enrollment and biological sample collection.

### Experimental design

All samples were collected from residents of Nam Phong and Ubolratana Districts, Khon Kaen Province, Northeast Thailand, under the Chronic Kidney Disease Northeastern Thailand (CKDNET) project (Fig. 1). Baseline samples (before-treatment group) were collected in July 2023 for screening to detect parasitic infections. Participants received ivermectin prescribed by a physician, and treatment was administered in October 2023. Follow-up samples (after-treatment group) were collected in March 2025, 17 months after ivermectin treatment. At baseline, intestinal parasite infections were diagnosed using established parasitological methods: the modified agar plate culture (mAPC) and the formalin-ethyl acetate concentration technique (FECT) (Yingklang et al., 2022), which allow for the detection of light-intensity infections. During follow-up, clearance was confirmed using both the mAPC and a molecular method, polymerase chain reaction (PCR), to verify the absence of the parasite (Nieves et al., 2024). Inclusion criteria for the present study were: infection with *Strongyloides stercoralis* alone (no other co-infecting intestinal parasites) and a diagnosis of early stage (stages 1–3) of CKD by nephrologists. According to questionnaire data, none of the patients had recently used or received medications or supplements that could affect immune function or metabolism, including antibiotics, antiparasitics, corticosteroids, immunomodulators, or biological agents such as recombinant hormones (insulin, erythropoietin) and probiotics.

**Figure 1:**
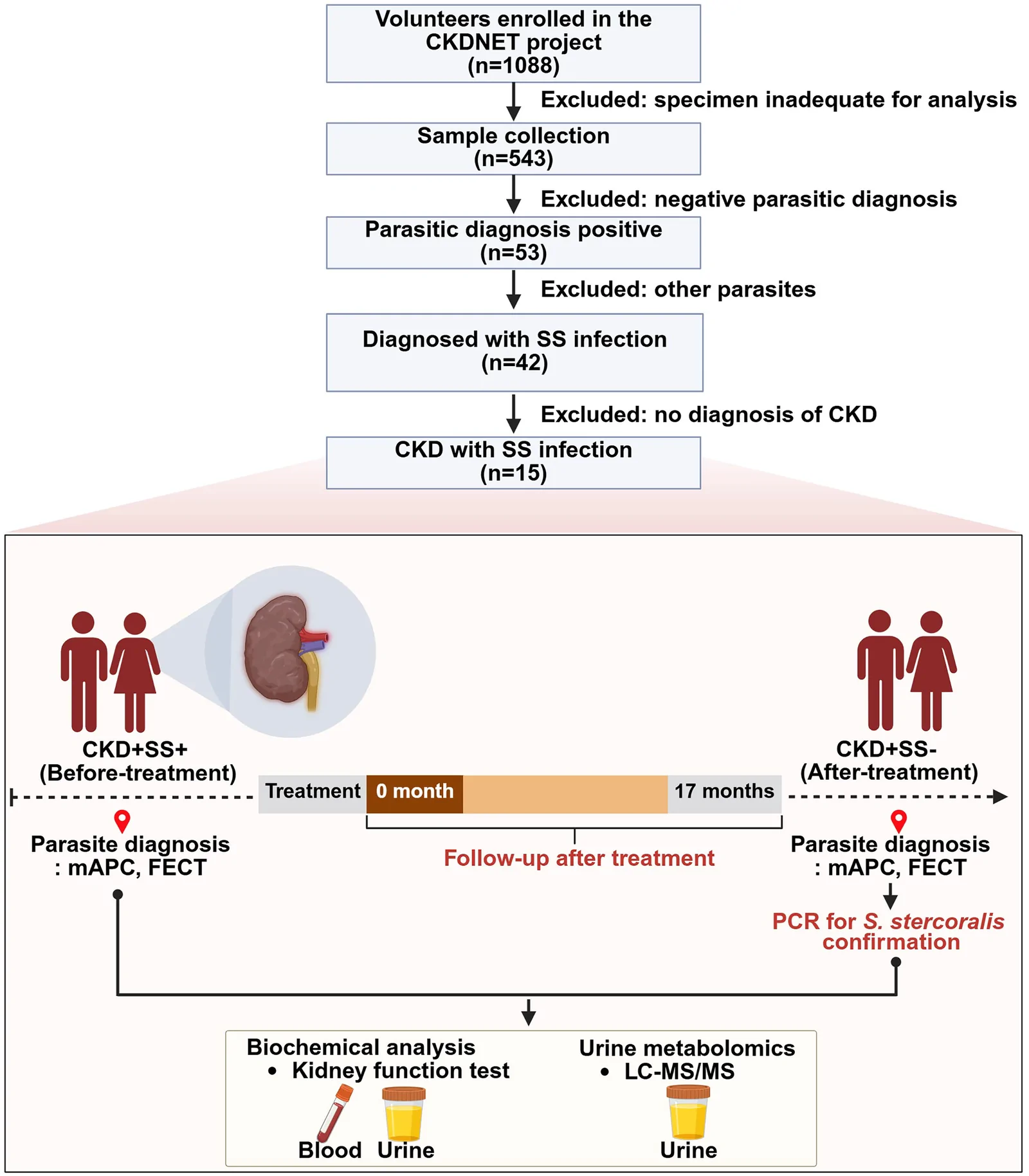
Flowchart of participant selection and study design. Abbreviations: SS, *S. stercoralis*; mAPC, modified agar plate culture; FECT, formalin-ethyl acetate concentration technique; CKD+SS+, CKD patient with *S. stercoralis* infection; CKD+SS−, CKD patient without *S. stercoralis* infection. Figure created with BioRender.com under license no. HC2A7JYO29.

### Sample collection and preparation

Venous blood samples were collected after an overnight fast of at least 8 h. Blood samples were centrifuged at 3,500 rpm for 10 min to separate serum, followed by aliquoting and immediate storage at −80 °C until analysis. Midstream first-morning urine samples were collected and centrifuged at 3,500 rpm for 10 min at 4 °C to remove cell debris. The resulting supernatant was stored at −80 °C until analysis to preserve sample integrity.

### Measurement of kidney function parameters in serum and urine

Blood and urine biochemical parameters related to kidney function, including fasting blood sugar (FBS), serum creatinine (sCr), urine creatinine (uCr), microalbumin (MA), and urine albumin-to-creatinine ratio (uACR), were measured using automated analyzers (Cobas 8000 Modular Analysis Series; Roche Diagnostics, Mannheim, Germany) at the clinical laboratory of Srinagarind Hospital, Faculty of Medicine, Khon Kaen University, Khon Kaen, Thailand.

### PCR to confirm *S. stercoralis* infection

Conventional PCR was applied to confirm post-treatment parasitological clearance from stool samples. Amplification of a species-specific target sequence of *S. stercoralis* (GenBank accession no. M84229.1) was conducted using previously published primers and PCR conditions (Iamrod et al., 2021). PCR products were analyzed by electrophoresis on a 2% agarose gel to confirm amplification and verify the expected amplicon size of 230 bp. Detailed primer sequences and amplification conditions are provided in Data S1.

### Sample preparation for urine metabolomics

Urine samples were thawed at 4 °C prior to mass spectrometry (MS) analysis. For sample preparation, 50 µL of each urine sample was mixed with 200 µL of 100% methanol containing 50 ng/mL sulfadimethoxine as the internal standard. The mixtures were vortexed thoroughly and centrifuged at 14,000 rpm for 10 min. The resulting supernatants were transferred to LC–MS vials for subsequent analysis.

### Untargeted urinary metabolomic analysis based on an LC/Q-TOF system

Metabolite separation was performed using a Poroshell 120 EC-C18 column (2.1 mm × 100 mm, 2.7 µm) maintained at 50 °C, with a 10 µL injection volume. The mobile phases consisted of 0.1% formic acid in water (mobile phase A) and 0.1% formic acid in acetonitrile (mobile phase B), delivered at 0.4 mL/min. The gradient elution was as follows: 0–0.5 min, 100% A; 0.5–10.5 min, 100–45% A; 10.5–12.5 min, 45–25% A; 12.5–14.0 min, 25–0% A; 14.0–17.0 min, 0% A; 17.0–17.5 min, 0–100% A; and 17.5–20.0 min, 100% A. Mass spectrometric detection was performed on an Agilent LC-QTOF 6545XT system (Agilent Technologies, Santa Clara, CA, USA) operated in high-resolution electrospray ionization (ESI) modes, both positive and negative. Parameters were set as follows: drying gas temperature 325 °C (flow rate 13 L/min), sheath gas temperature 275 °C (flow rate 12 L/min), nebulizer pressure 45 psi, and capillary voltages of 4,000 V (positive mode) and 3,000 V (negative mode). The isotope width was set to 1.3 m/z. Full-scan MS1 and MS2 data were collected over mass range of m/z 40–1,700 and m/z 25–1,000, respectively. Collision energies were set to 20 eV (positive mode) and 10 eV (negative mode). Data acquisition was performed at a rate of 3.35 spectra/s with a maximum of 10 precursors per cycle and a precursor threshold of 5,000 counts. The retention time threshold was set to 0.001%. Continuous mass calibration was achieved using reference ions at m/z 121.0509 and 922.0098 (positive mode), and m/z 112.9856 and 1,033.9881 (negative mode).

### Data processing and feature extraction

Raw LC-MS data were processed using MS-DIAL software version 5.3. Peak alignment was performed using one of the quality-control (QC) samples as a reference. Peak intensities were normalized to the internal standard sulfadimethoxine and adjusted using the locally weighted scatterplot smoothing method. Mass exclusion was applied to minimize analytical variation. To reduce background interference, specific mass features were excluded from analysis, including m/z 121.0509 and 922.0098 (positive mode) and m/z 112.9856, 119.0363, 966.0007, and 1,033.9881 (negative mode). Adduct forms were assigned as [M+H]^+^ in positive mode and [M+H]^+^ in negative mode. Metabolite identification was performed by annotating MS/MS spectra against the MS-DIAL ESI (±) MS/MS library of authentic standards. To ensure data quality, only features meeting the following criteria were retained: retention time between 0.2 and 18 min, mass error <±20 ppm, Pearson correlation coefficient ≥0.70, and coefficient of variation (%CV) among QC samples <30%. An identification score of ≥0.70 was required and unidentified features were excluded from further analysis.

### Urine metabolomic profiling analysis

All analyses were conducted using R software (version 4.5.1; R Core Team, 2025) within the RStudio environment (version 2023.09.1; Posit, Boston, MA, USA). Prevalence filtering was applied to exclude metabolites detected in less than 30% of all samples from further analysis. For metabolite intensity containing zero, missing, or non-finite values, replacement was performed using half of the smallest positive value detected for that metabolite (half-min imputation) with the dplyr package (version 1.1.4). Log_2_ transformation and auto scaling were applied prior to multivariate analyses, including principal component analysis (PCA) and partial least-squares discriminant analysis (PLS-DA). In contrast, Log_2_ fold changes (Log_2_FC) were calculated directly from the imputed data. PCA was performed using the prcomp() function (stats package; Stats Perform, London, UK). PLS-DA and variable importance in projection (VIP) score calculation were performed using the plsda() and vip() functions, respectively, from mixOmics package (version 6.32.0). Model performance was assessed using the area under the receiver operating characteristic curve (AUC), estimated by 5-fold cross-validation with 10 repeats. Statistical significance was evaluated by permutation testing (*n* = 1,000) using the perf() function in the mixOmics R package. Paired Wilcoxon signed-rank tests (wilcox.test function) were applied to compare metabolite intensities in samples before and after treatment, with effect sizes expressed as the median log_2_FC, calculated as log_2_(after/before). *p*-values were adjusted using the Benjamini-Hochberg method with the p.adjust() function from the stats package. Metabolites with *p*-value < 0.05, |log_2_FC| > 1, and VIP score > 1 were considered significant. Candidate metabolites for correlation analyses were selected using stricter thresholds: *p*-value < 0.01, |log_2_FC| > 1, and VIP score > 2. Spearman correlations between these metabolites and kidney function test parameters were computed using the corr.test() function from the psych package (version 2.5.6). All figures, including PCA and PLS-DA score plots, volcano plots, boxplots, correlation heatmaps, and scatter plots, were visualized using ggplot2 (version 3.5.2). Significant metabolites were submitted to MetaboAnalyst version 6.0 (https://www.metaboanalyst.ca/, accessed 1 August 2025) for enrichment and pathway analyses using the Small Molecule Pathway Database (SMPDB) and Kyoto Encyclopedia of Genes and Genomes (KEGG). Pathways with *p*-value < 0.05 were considered enriched.

### Statistical analysis

Statistical analyses were performed using GraphPad Prism (version 8; GraphPad Software, San Diego, CA, USA) and IBM SPSS Statistics (version 21; IBM Corp., Armonk, NY, USA). The distribution of each biochemical variable was assessed using the Shapiro-Wilk normality test. For paired measurements, normally distributed variables were compared using the paired t-test, whereas non-normally distributed variables were compared using the paired Wilcoxon signed-rank test. A two-sided *p*-value < 0.05 was considered statistically significant. Data are presented as median and interquartile range (IQR).

## Results

Among 1,088 individuals screened, 15 patients with early-stage (stages 1–3) CKD and confirmed *Strongyloides stercoralis* infection met the inclusion criteria and were enrolled in the study. The baseline demographic and clinical characteristics of the participants are summarized in Table 1. Post-treatment assessments confirming parasite clearance in all patients are presented in Fig. S1.

**Table 1:** Baseline characteristics of CKD patients with *S. stercoralis* infection included in the metabolomics study.

Characteristics	Value (*n* = 15)
Age, years (mean ± SD)	65.27 ± 9.81
Gender	
Male (*n*, %)	7, 46.67
Female (*n*, %)	8, 53.33
Body mass index (BMI), kg/m^2^ (mean ± SD)	24.11 ± 7.29
CKD stage	
Stage 1 (*n*, %)	3, 20
Stage 2 (*n*, %)	9, 60
Stage 3 (*n*, %)	3, 20

**Note:**

CKD, chronic kidney disease; *S. stercoralis*, *Strongyloides stercoralis*.

### Treatment-induced changes in biochemical parameters

Significant changes in biochemical parameters were observed at 17 months post-treatment for strongyloidiasis (Figs. 2A–2F), with bar graphs showing individual paired data points presented in Figs. S2A–S2F. FBS was significantly lower following treatment than at baseline (*p* < 0.05). sCr and uCr levels declined (*p* < 0.05), while eGFR increased (*p* < 0.05). In addition, kidney damage markers, including microalbumin (MA) and uACR, were also markedly reduced (*p* < 0.05). These findings suggest an association between parasite clearance and favorable changes in glycemic and renal biomarkers in patients with CKD. A full list of these data is provided in Table S1.

**Figure 2:**
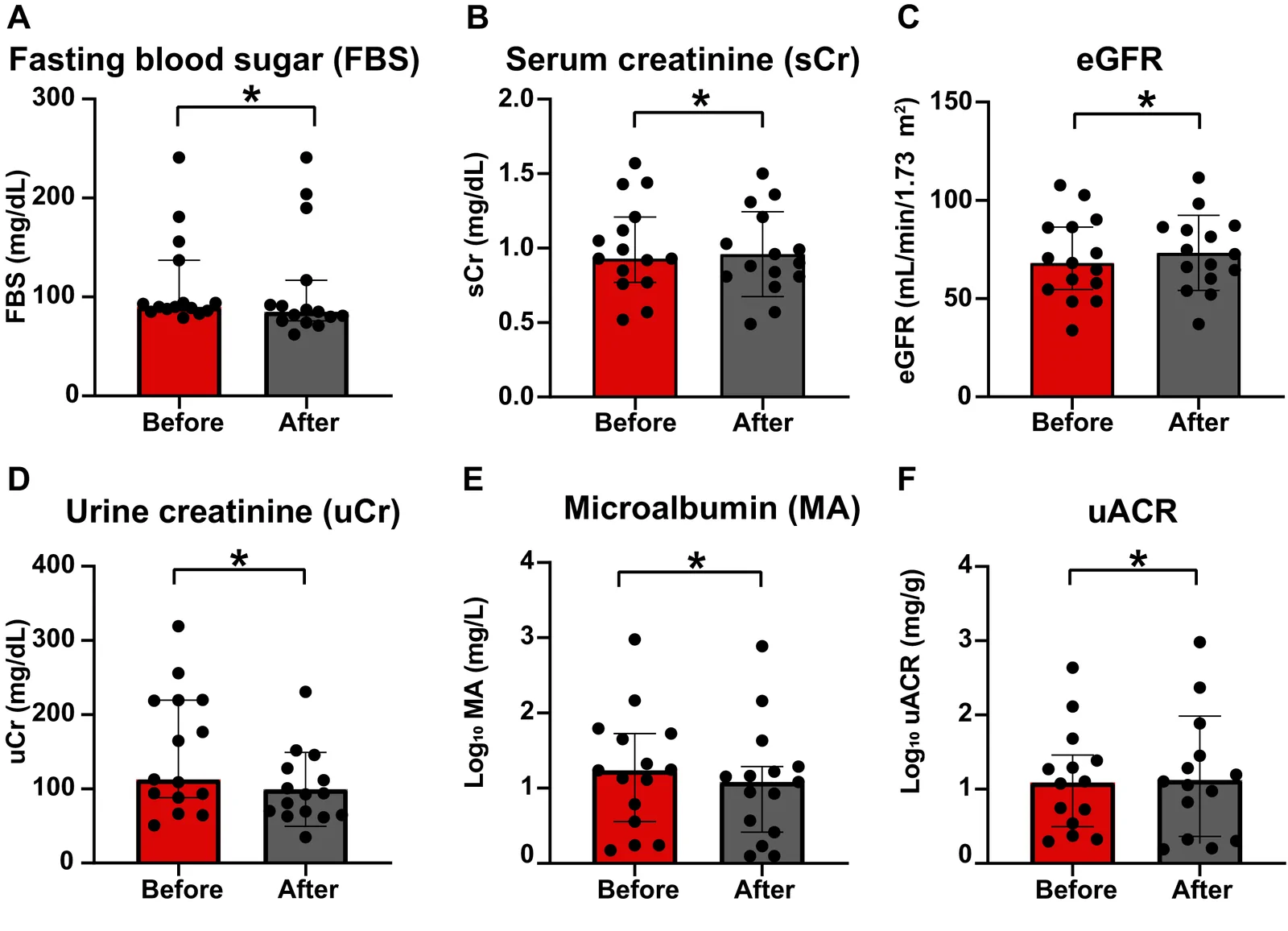
Effect of *S. stercoralis* treatment on biochemical parameters in CKD patients. Red bars represent before-treatment, and gray bars represent after-treatment. Parameters include (A) fasting blood sugar (FBS), (B) serum creatinine (sCr), (C) estimated glomerular filtration rate (eGFR), (D) urine creatinine (uCr), (E) microalbumin (MA), and (F) urine albumin-to-creatinine ratio (uACR). *Indicates *p* < 0.05.

### Multivariate analysis of urinary metabolomics changes following treatment

Unsupervised PCA and supervised PLS-DA were performed to evaluate global urinary metabolomics variations between the pre- and post-treatment states in both negative and positive ionization modes. The PCA (Figs. 3A, 3B) showed substantial overlap between the pre- and post-treatment samples in both modes, indicating no clear global metabolomic separation. Consistent with these findings, PLS-DA (Figs. 3C, 3D) did not reveal significant global metabolomic separation between pre- and post-treatment samples in the negative mode (AUC = 0.64; *p* = 0.16) and positive mode (AUC = 0.24; *p* = 0.97).

**Figure 3:**
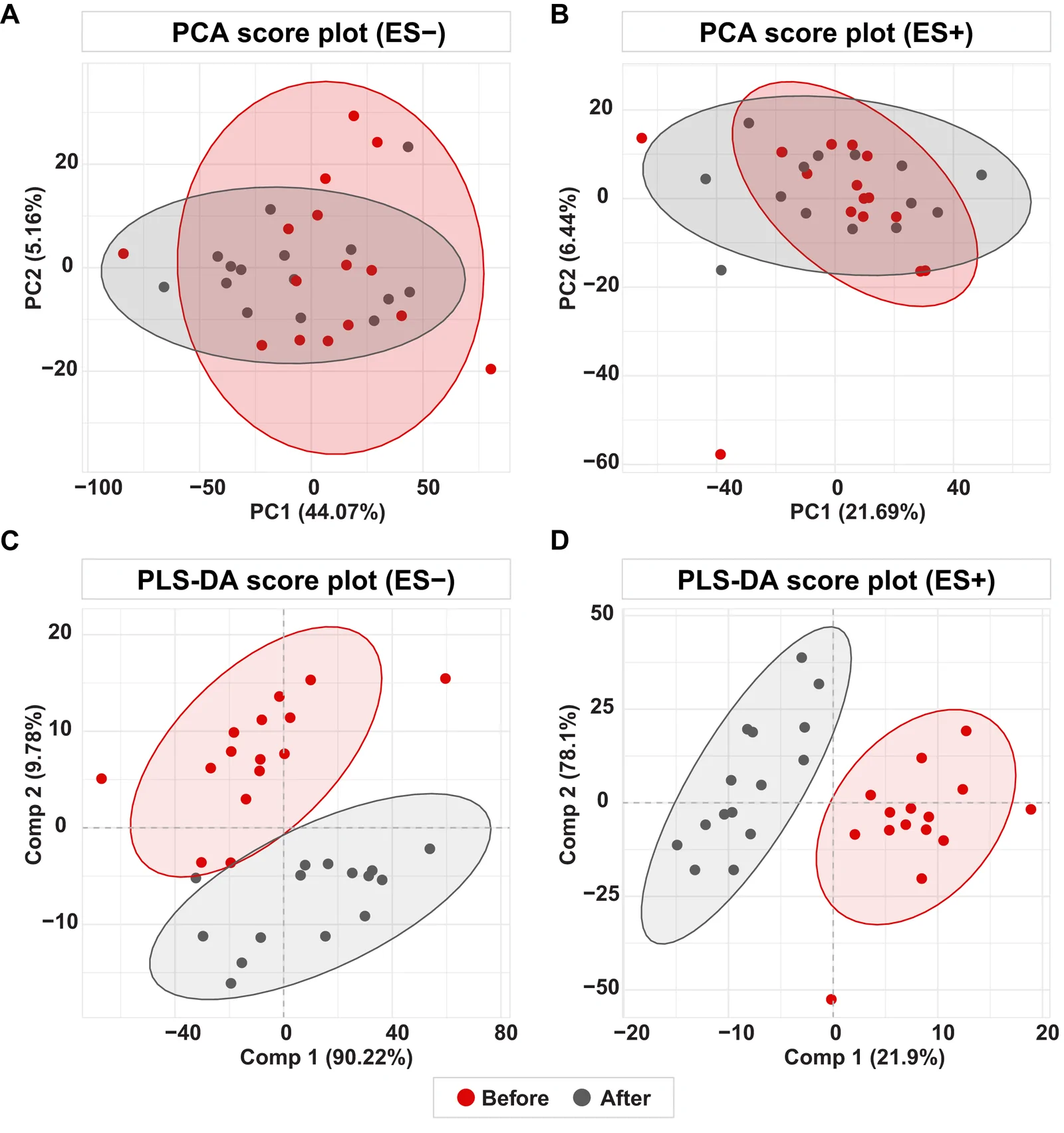
PCA and PLS-DA score plots showing separation between before-treatment (red) and after-treatment (gray) *S. stercoralis* groups in CKD patients. PCA score plots: (A) negative ionization mode (ES−) and (B) positive ionization mode (ES+). PLS-DA score plots: (C) ES− mode and (D) ES+ mode.

### Identification of post-treatment differential urine metabolite patterns

Untargeted metabolomics revealed alterations in urinary metabolic profiles in CKD patients following *S. stercoralis* treatment. In negative (ES−) mode, 71 metabolites were significantly altered (*p* < 0.05, |log_2_FC| > 1, VIP scores > 1), comprising 68 decreases and 3 increases (Fig. 4A). In positive (ES+) mode, 20 metabolites were significantly changed, including 15 decreases and 5 increases (Fig. 4B). Across both ionization modes, the overall alteration patterns were similar, with most significant changes representing decreased metabolite abundance after therapy, suggesting treatment-associated metabolic changes. All detected metabolites are listed in Table S2.

**Figure 4:**
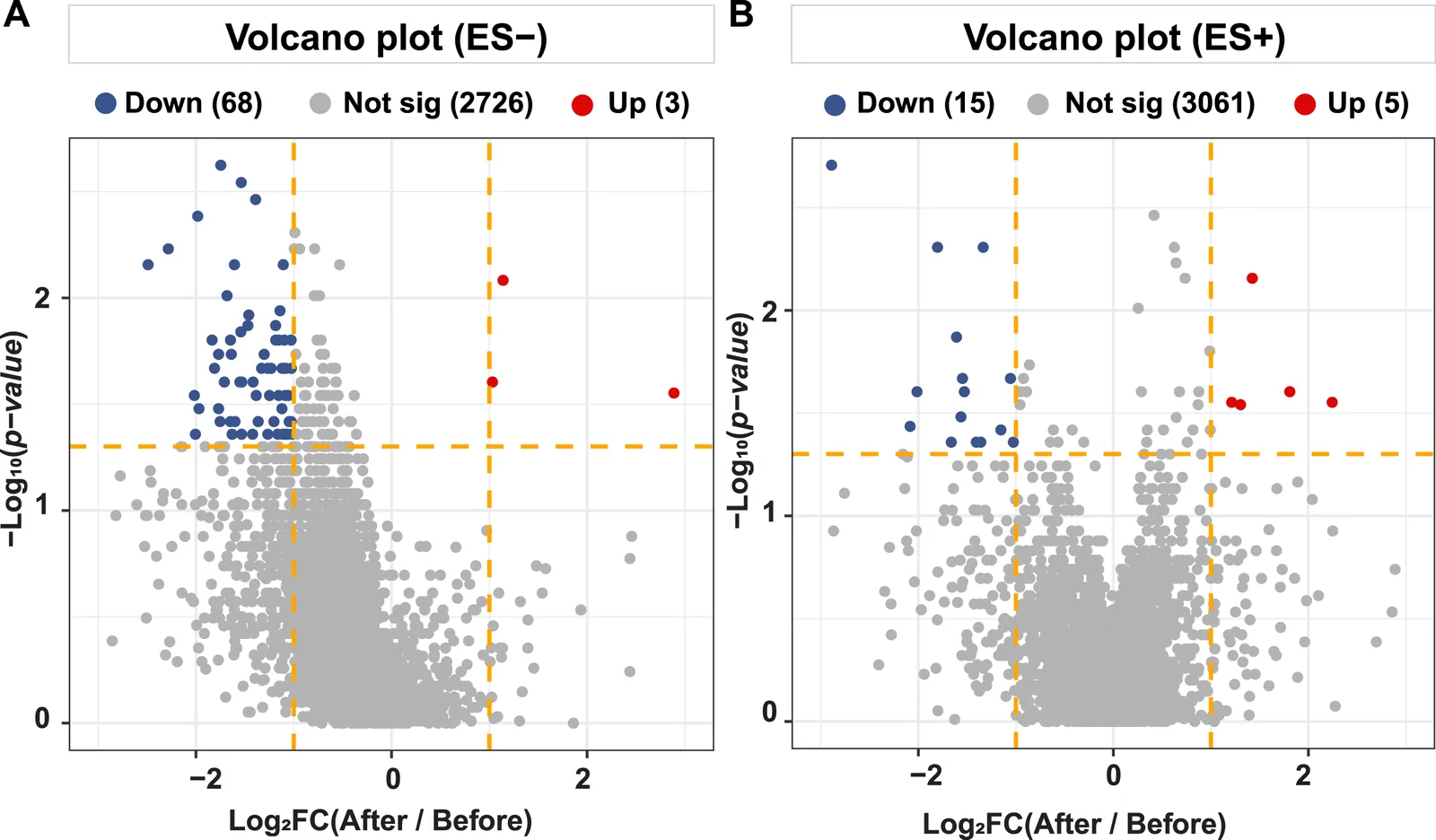
Volcano plots illustrate patterns of urine metabolite alterations following therapy. (A) Negative ionization mode (ES−) and (B) positive ionization mode (ES+). Blue dots indicate significantly downregulated metabolites, red dots indicate significantly upregulated metabolites, and grey dots represent non-significant changes at *p* < 0.05 and |log_2_ FC| > 1.

Applying stricter selection thresholds (*p* < 0.01, |log_2_FC| > 1, VIP > 2), nine candidate metabolites were identified in ES− mode: gluconic acid-γ-lactone, C_5_H_10_O_5_, isoeugenitol, phenyl acetyl aspartic acid, chrysophanol, sedoheptulose-7-phosphate, neochlorogenic acid, paulownin, and 1-O-galloyl-6-O-cinnamoylglucose (Fig. 5). In ES+ mode, four candidates were identified: 1H-indole-3-carboxamide, carbendazim, ascorbic acid, and C_15_H_20_O_8_ (Fig. 6). The consistent changes observed in these metabolites suggest that they may warrant further investigation as candidate markers of metabolic responses following *S. stercoralis* clearance in patients with CKD.

**Figure 5:**
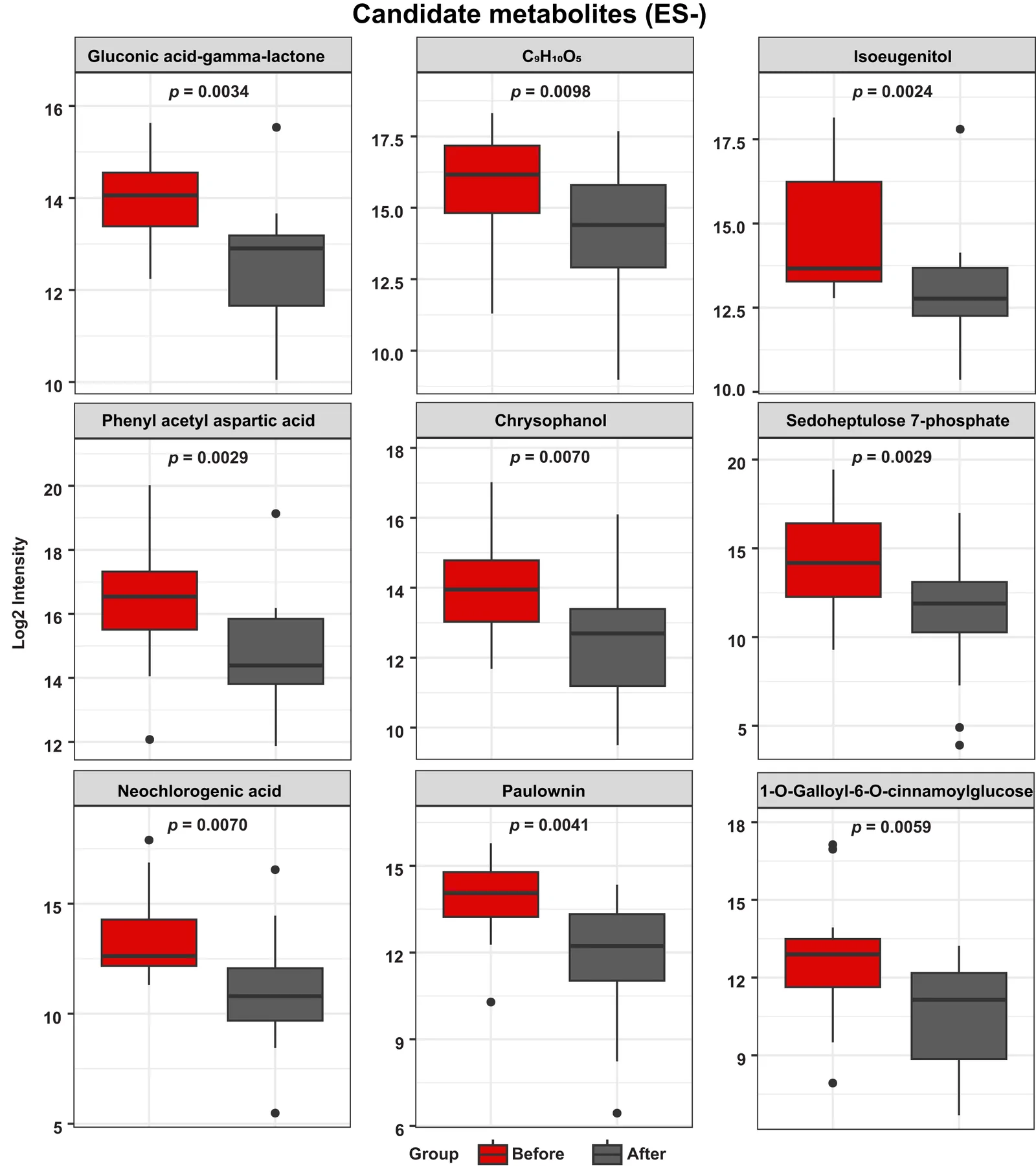
Box plots of differences in abundance of candidate urine metabolites identified in negative ionization mode (ES−). Before-treatment (red) and after-treatment (grey) conditions.

**Figure 6:**
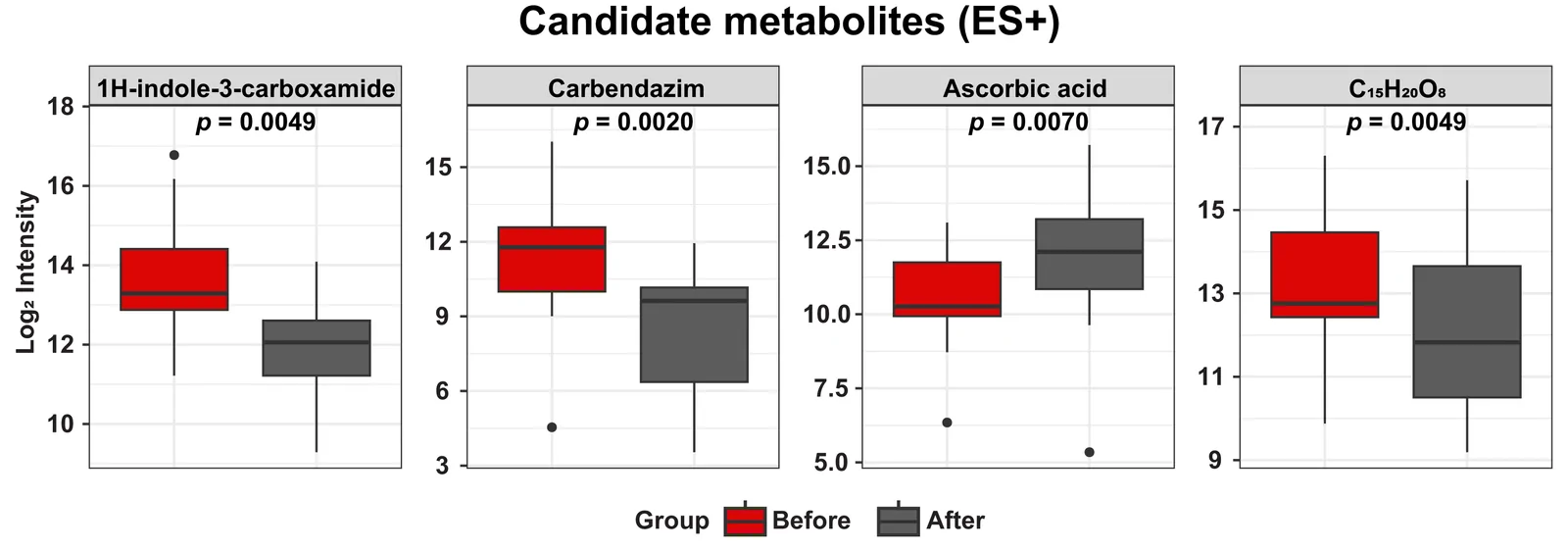
Box plots of differences in abundance of candidate urine metabolites identified in positive ionization mode (ES+). Before-treatment (red) and after-treatment (grey) conditions.

### Correlation between candidate urine metabolites and kidney function parameters

Heatmap analysis revealed changes in Spearman’s correlations between metabolites and kidney function markers after *S. stercoralis* treatment, showing absolute correlation coefficients (|r|) of up to 0.61 (Fig. 7), with the complete set of correlation coefficients provided in Table S3. Notably, in negative ionization mode after treatment, 1-O-galloyl-6-O-cinnamoylglucose (Fig. 8A) and sedoheptulose-7-phosphate (S7P) (Fig. 8B) showed a significant negative correlation with urine creatinine (r = −0.61, *p* = 0.016) and serum creatinine (r = −0.54, *p* = 0.039), respectively. As urine and serum creatinine levels decreased after treatment, the observed decreases in these metabolites suggest their potential relevance as markers of treatment-associated changes in kidney function. In addition, in positive ionization mode before treatment, ascorbic acid (Fig. 8C) exhibited an inverse correlation with uCr (r = −0.53, *p* = 0.044).

**Figure 7:**
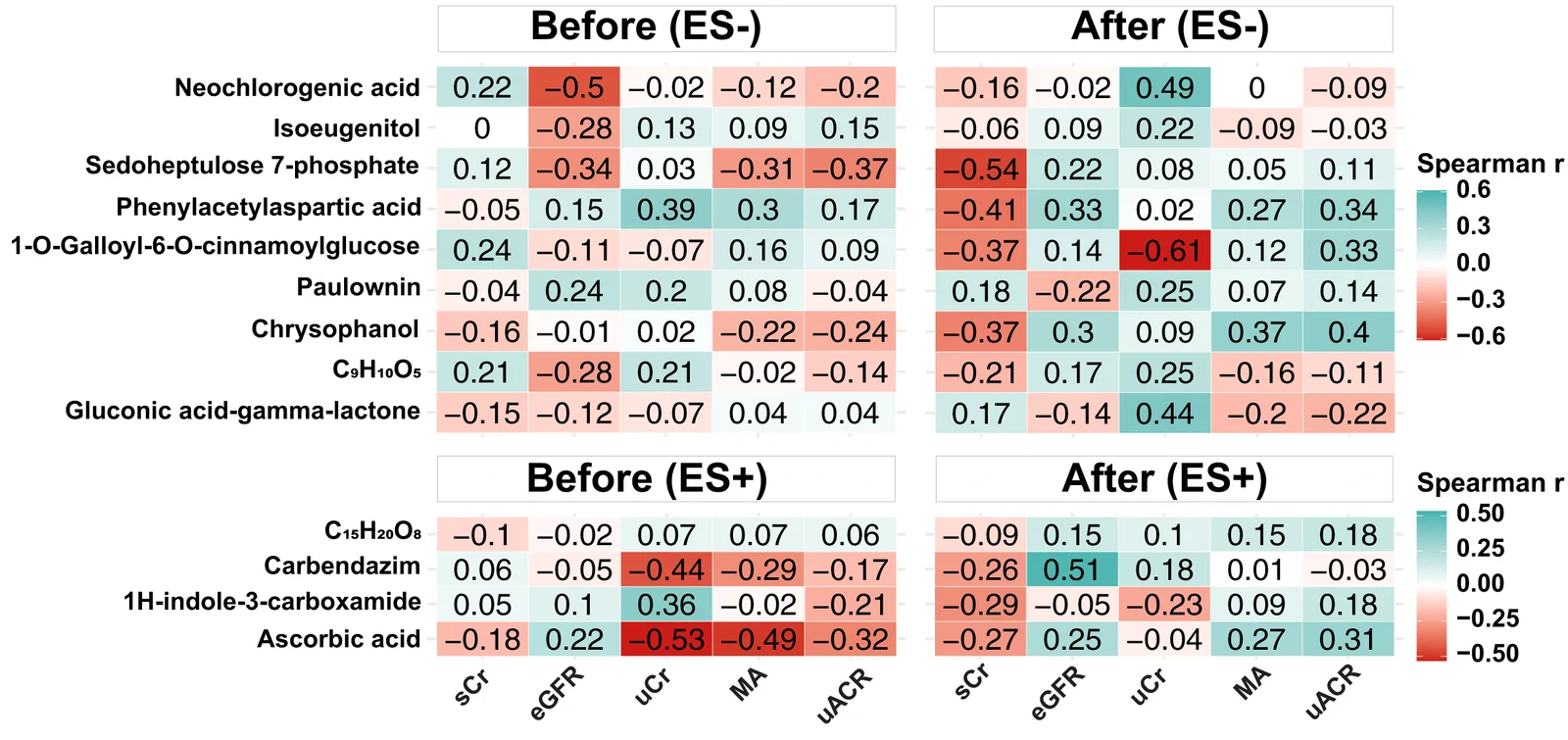
Heatmap of Spearman’s correlation coefficients between candidate urine metabolites from negative (ES−) and positive (ES+) modes and kidney function parameters. Abbreviations: serum creatinine (sCr), estimated glomerular filtration rate (eGFR), urine creatinine (uCr), microalbumin (MA), and urine albumin-to-creatinine ratio (uACR). The color indicates the direction of the correlation (green = positive, red = negative) and the color intensity indicates the relative strength of any correlation.

**Figure 8:**
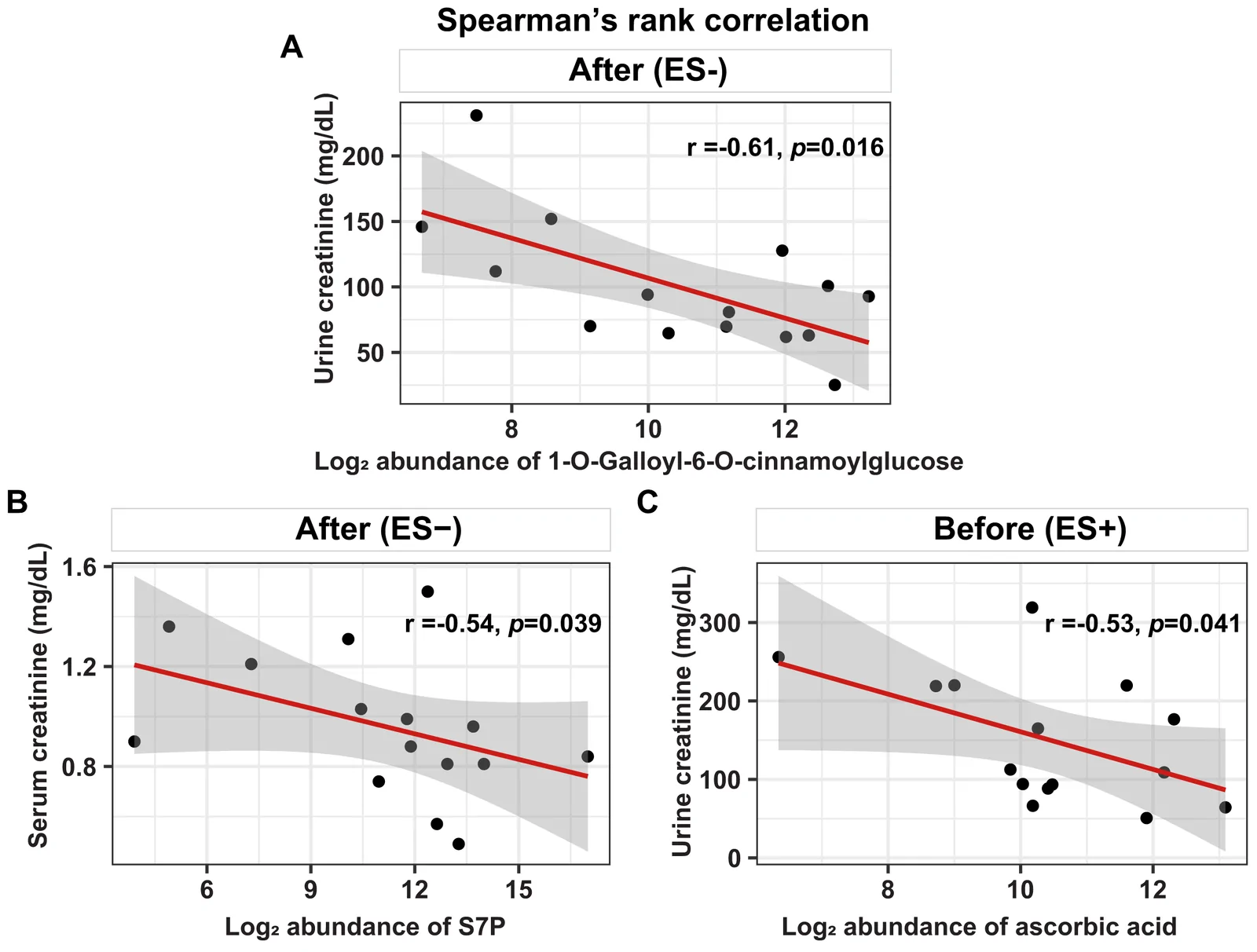
Scatter plots show significant correlations between candidate urine metabolites and kidney function parameters. The X-axis represents the log₂ abundance of each metabolite, and the Y-axis represents the corresponding biochemical parameter. (A, B) Negative ionization mode (ES−) and (C) positive ionization mode (ES+).

### Identification of pathway enrichment from significant negative mode urine metabolite profiles

Pathway enrichment analysis of significant metabolites detected in negative ionization mode (Fig. 9A and Table S4) identified pathways related to carbohydrate metabolism, amino acid metabolism, and energy/redox pathways, which may reflect alterations in metabolic processes associated with oxidative stress. The SMPDB (Fig. 9B and Table S5) and KEGG (Fig. 9C and Table S6) analyses identified key pathways such as glycine/serine metabolism, tyrosine metabolism, and starch/sucrose metabolism, suggesting that metabolites altered following antiparasitic treatment are associated with changes in amino acid, carbohydrate, and redox-related metabolic processes in patients with CKD.

**Figure 9:**
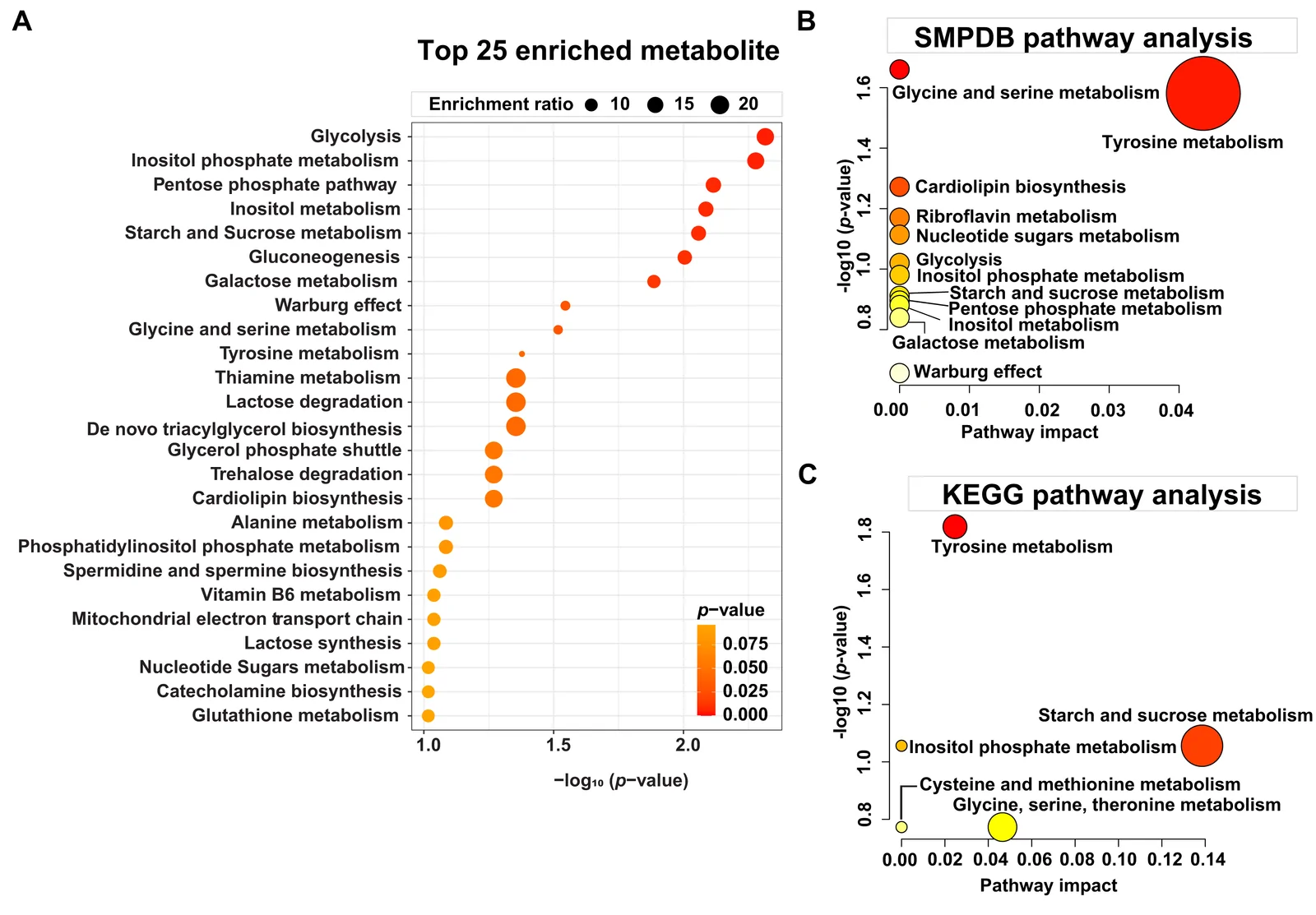
Pathway enrichment analysis of significant negative ionization mode urine metabolites. (A) Bubble plot of the top 25 enriched metabolic pathways ranked by −log_10_ (*p*-value) and enrichment ratio. Bubble size indicates the enrichment ratio, bubble color represents the *p*-value, with darker colors denoting higher statistical significance. (B) SMPDB pathway analysis and (C) KEGG pathway analysis.

## Discussion

This study is to provide metabolomic evidence that treating *Strongyloides stercoralis* infection in patients with CKD can significantly improve both clinical and metabolic markers of renal function. By integrating clinical biochemistry with untargeted metabolomics, we revealed that parasite clearance leads to a broad downregulation of host metabolites, suggesting the infection imposes a substantial metabolic burden that is alleviated by treatment. This was accompanied by marked improvements in renal function, including reduced sCr and albuminuria, and increased eGFR. These results strongly suggest that *S. stercoralis* infection is an active contributor to metabolic dysregulation. Additionally, our findings have uncovered distinct metabolic signatures of infection and recovery, identifying novel candidate biomarkers for monitoring CKD-associated strongyloidiasis. Our longitudinal results strongly support and extend previous cross-sectional findings indicating that *S. stercoralis* infection is associated with poorer renal parameters, including reduced eGFR and elevated uACR (Yingklang et al., 2022). Importantly, our intervention study suggests that parasite clearance is associated with improvements in kidney function in patients with early-stage CKD and *S. stercoralis* infection. This observed improvement in renal function suggests that parasite eradication may alleviate underlying pathologies, such as the systemic inflammation, oxidative stress, and gut microbiome alterations previously linked to *S. stercoralis* infections and CKD progression (Hai et al., 2022; Rajamanickam et al., 2020; Tran et al., 2023b). However, our findings on glycemic control markedly differ from previous reports. We observed that parasite clearance was associated with significantly improved FBS levels, implying that *S. stercoralis* infection may contribute to hyperglycemia. This contrasts with reports of an inverse correlation between infection and type 2 diabetes (Yingklang et al., 2022) and with findings showing no association with diabetes (Salvador et al., 2023). This inconsistency likely reflects key differences in study design (our longitudinal follow-up compared with their cross-sectional approach) as well as our focus on a patient population with only early stages of CKD. The effect we observed may be mediated by chronic parasite-induced inflammation, which is known to contribute to insulin resistance (Dai et al., 2022). Given that hyperglycemia is a major risk factor for CKD progression (Kumar et al., 2023), the enhanced glycemic control following parasite clearance may represent an additional mechanism protecting renal function in these patients.

Urinary metabolite profiling before and after treatment confirms that *S. stercoralis* infection imposes a distinct systemic metabolic burden on the host. Similar findings have been reported in other helminth infections, including *Schistosoma* and *Trichinella*, which also disrupt host lipid, amino acid, and glucose metabolism (Chienwichai et al., 2023; Rodrigues et al., 2025). Elimination of the parasite thus attenuates these disturbances and promotes restoration of the host metabolome toward a regulated, homeostatic state. Several metabolites that decreased after treatment, such as gluconic acid-γ-lactone, sedoheptulose-7-phosphate (S7P), neochlorogenic acid, and chrysophanol, have previously been implicated in oxidative stress and renal injury pathways (Cui et al., 2021; Gu et al., 2022; Tsai et al., 2025). Their alteration likely reflects systemic metabolic disturbances and elevated oxidative stress associated with CKD. Gluconic acid-γ-lactone, a precursor of gluconic acid, is associated with the deterioration of renal function and oxidative stress in end-stage CKD (Kimura et al., 2016). S7P, an intermediate of the pentose phosphate pathway, accumulates under oxidative stress, reflecting an increased cellular demand for NADPH, a critical molecule for antioxidant defense (Pawłowska et al., 2024). The post-treatment reduction in these metabolites, combined with the negative correlation of S7P with serum creatinine, indicates a reduced oxidative burden and a return towards metabolic homeostasis. Similarly, decreased levels of neochlorogenic acid and chrysophanol, known for their roles in modulating inflammation and fibrosis (Gu et al., 2022; Tsai et al., 2025), indicate reduced activation of inflammatory and fibrotic pathways in the kidney. Notably, the co-occurrence of the xenobiotic fungicide carbendazim and *S. stercoralis* likely results from environmental co-exposure in high-risk settings, where carbendazim is a known pollutant of food, soil, and water (Singh et al., 2016) and can enter the body *via* ingestion or dermal contact (Zhou et al., 2023). However, the importance finding is that high pre-treatment carbendazim accumulation, which declined after parasite eradication, strongly implies the infection itself impaired xenobiotic clearance. This impairment is biologically likely, as *S. stercoralis*-induced inflammation and gut dysbiosis can suppress hepatic (*e.g*., cytochrome P450) and microbial detoxification pathways. This parasite burden likely compounded the compromised excretory capacity in these CKD patients (Vaziri, 2012), and parasite eradication appears to have restored this clearance capacity by removing the inflammatory suppression. Additionally, some of these key metabolites, including ascorbic acid and 1-O-galloyl-6-O-cinnamoylglucose, are primarily derived from dietary sources (Berretta et al., 2020; Villegas-Aguilar et al., 2020). *Strongyloides stercoralis* infection may disrupt gut microbiota, alter the microbial proteome (Tran et al., 2023a) and bacterial amino-acid metabolism in early-stage CKD (Tran et al., 2023b), leading to alteration of these urinary metabolites. The gut microbiome metabolizes a wide range of host- and diet-derived compounds, significantly influencing human health (Kim, Seo & Kweon, 2024). Ascorbic acid (vitamin C) was the only metabolite that increased after treatment, consistent with its well-established antioxidant and renoprotective functions (Karahan, Afsar & Kanbay, 2018; Wei et al., 2024). Its increase and inverse correlation with urinary creatinine indicate restoration of antioxidant defenses and reduced oxidative burden. Strongyloidiasis may worsen outcomes in CKD patients and appears to be associated with vitamin C deficiency. This suggests that the heightened oxidative stress from the infection depletes the body’s antioxidant defenses, leaving the kidneys vulnerable to further injury (Eryılmaz Pehlivan, 2017). Healthy kidneys can excrete excess vitamin C by filtering it out in urine, as vitamin C is water-soluble (Takahashi et al., 2011). However, in individuals with kidney disease or impaired kidney function, the kidneys cannot efficiently excrete the excess vitamin C. Patients with renal dysfunction, particularly in stages 4–5, have inadequate or deficient levels of plasma vitamin C (Doorenbos et al., 2025). Moreover, elevated serum vitamin C concentrations are associated with a reduced risk of CKD (Wang et al., 2024a), suggesting that carefully increasing serum vitamin C levels may help reduce the risk of developing CKD. Our results underscore the clinical importance of assessing vitamin C levels in the CKD population and represent a potential target for intervention (Chazot, Steiber & Kopple, 2023). Taken together, the metabolite–disease associations strengthen the interpretation that *S. stercoralis* infection may contribute to kidney injury through interconnected mechanisms involving oxidative stress, inflammation, impaired metabolic clearance, and gut-derived metabolites (Anandh & Saxena, 2024; Hobby et al., 2019).

Recent advances in metabolomics have demonstrated the utility of metabolic profiling for biomarker discovery in kidney diseases (Wang et al., 2024b; Xu et al., 2024). Consistent with these findings, our study suggests that urinary metabolomics can identify treatment-associated metabolic changes and potential biomarkers of recovery, including S7P and ascorbic acid, in CKD patients with *S. stercoralis* infection. Pathway enrichment analysis supported these findings, revealing significant disturbances in carbohydrate and amino acid metabolism, consistent with previous report demonstrating that parasitic infections can profoundly alter host homeostasis at a molecular level (Rajamanickam et al., 2019). Among these, glycine/serine metabolism, tyrosine metabolism, and glycolysis were most prominently affected. Disruption of glycine/serine metabolism is particularly significant, as this pathway is essential for cellular defense against oxidative stress. Notably, glycine functions as a rate-limiting precursor in the synthesis of glutathione (GSH), the key intracellular antioxidant molecule (Alves et al., 2019). Chronic parasitic infections are known to induce inflammation and oxidative stress (Rajković et al., 2025), both through host immune activation (Sorci & Faivre, 2009) and parasite-derived excretory-secretory products (Chulanetra & Chaicumpa, 2021). Perturbation of glycolysis was also consistent with host metabolic reprogramming to meet the elevated energy demands during infection (Bohara & Kumar, 2026).

This study has several limitations, including the small sample size and the lack of a control group of CKD patients without *S. stercoralis* infection, which prevents us from distinguishing infection-specific metabolic signatures from general CKD-related metabolic alterations. The limited sample size may also increase the influence of inter-individual variability and restrict the generalizability of the findings. In addition, data were collected at only two time points (baseline and 17-month follow-up), limiting the assessment of temporal trends and increasing susceptibility to unmeasured confounding factors. Therefore, the observed associations between *S. stercoralis* infection, kidney function, and metabolomic profiles should be interpreted with caution. Further studies with larger cohorts, appropriate control groups, and longitudinal sampling are needed to validate these findings.

## Conclusions

In conclusion, treatment of *S. stercoralis* infection in patients with early-stage CKD was associated with improvements in clinical kidney function and changes in metabolomic profiles. Changes in carbohydrate and amino acid metabolism were observed following antiparasitic treatment, including pathways previously associated with oxidative stress. Specific metabolites, including sedoheptulose-7-phosphate and ascorbic acid, are potential biomarkers of metabolic response. These findings suggest that *S. stercoralis* infection was associated with metabolic alterations in patients with CKD and support further investigation of the potential role of strongyloidiasis screening in this population. Future studies with larger cohorts and appropriate control groups are warranted to validate these biomarkers and further evaluate the relationship between antiparasitic treatment and long-term renal outcomes.

## Supplemental Information

10.7717/peerj.21832/supp-1 Supplemental Information 1 PCR confirmation of *Strongyloides stercoralis* clearance after treatment. 2% Agarose gel electrophoresis of PCR products. (A) Lanes include M, PC, samples 1–8, and NC. (B) Lanes include M, PC, samples 9–15, and NC. M = DNA marker; PC = pooled positive cases; NC = negative control (DI water); lanes 1–15 represent samples 1 to 15, respectively.

10.7717/peerj.21832/supp-2 Supplemental Information 2 Bar graphs with individual data points for biochemical parameters. Parameters include (A) fasting blood sugar (FBS), (B) serum creatinine (sCr), (C) estimated glomerular filtration rate (eGFR), (D) urine creatinine (uCr), (E) microalbumin (MA), and (F) urinary albumin-to-creatinine ratio (uACR)

10.7717/peerj.21832/supp-3 Supplemental Information 3 PCR primer sequences and amplification conditions used for confirmation of clearance of *Strongyloides stercoralis* infection.

10.7717/peerj.21832/supp-4 Supplemental Information 4 Supplemental Tables.

10.7717/peerj.21832/supp-5 Supplemental Information 5 Codebook.

## Additional Information and Declarations

### Data Availability

The following information was supplied regarding data availability:

The data is available in the Supplemental Files.

The metabolomics dataset is available at: http://dx.doi.org/10.21228/M89R9P (Project ID: PR002602).
