# Supplementary material for: Clearance of Strongyloides stercoralis infection using ivermectin improves kidney function and modulates untargeted urinary metabolomic profile in patients with early-stage chronic kidney disease
Source: PeerJ. 2026 Oct 29;14:e21832. doi: 10.7717/peerj.21832 (PMC13724049; doi:10.7717/peerj.21832)
Supplement: Supplementary file 1 [file peerj-14-21832-s001.pdf]

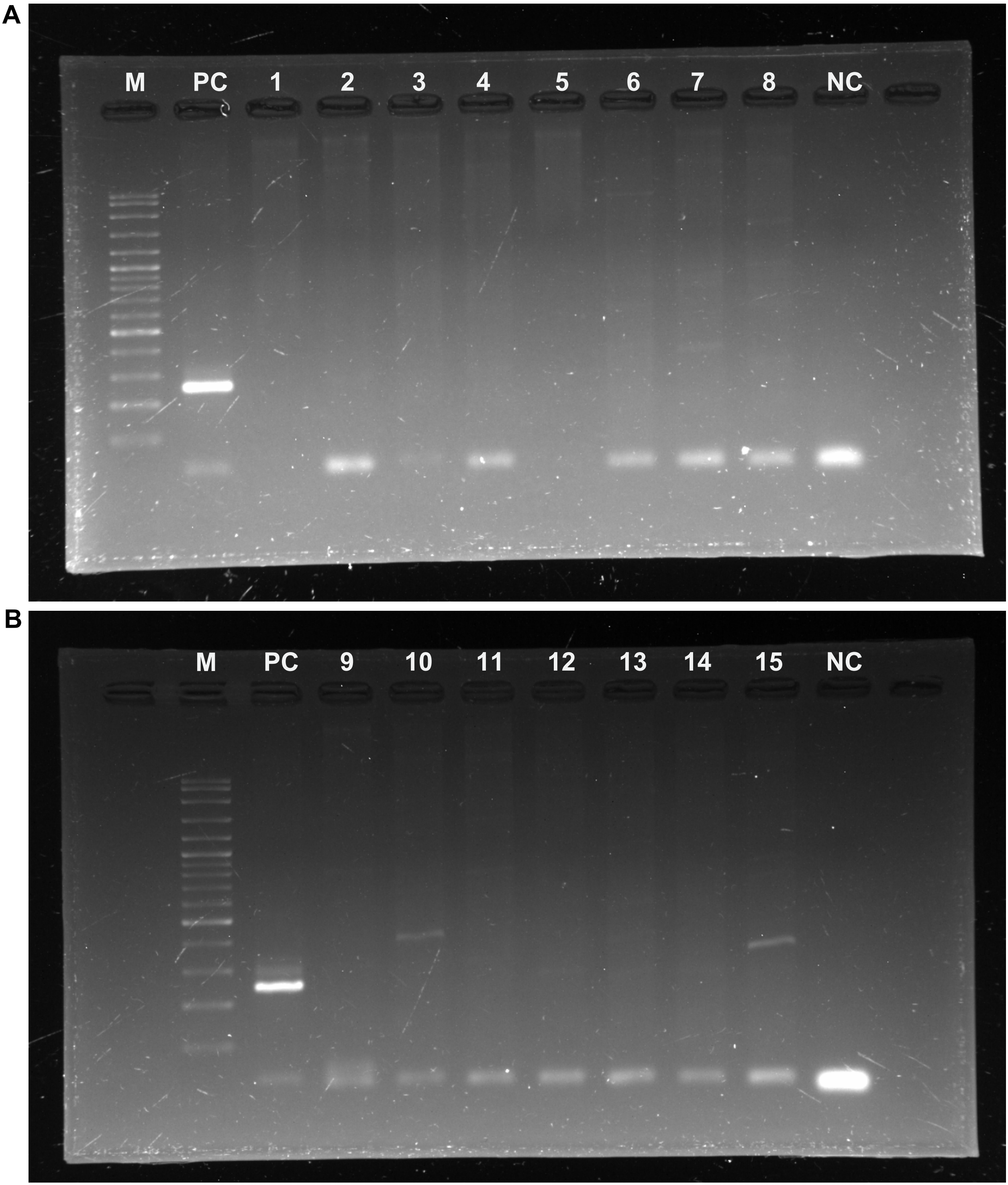
A
M
PC
1
2
3
4
5
6
7
8
NC
B
M
PC
9
10
11
12
13
14
15
NC
