# Supplementary material for: Clearance of Strongyloides stercoralis infection using ivermectin improves kidney function and modulates untargeted urinary metabolomic profile in patients with early-stage chronic kidney disease
Source: PeerJ. 2026 Oct 29;14:e21832. doi: 10.7717/peerj.21832 (PMC13724049; doi:10.7717/peerj.21832)
Supplement: Supplementary file 2 [file peerj-14-21832-s002.pdf]

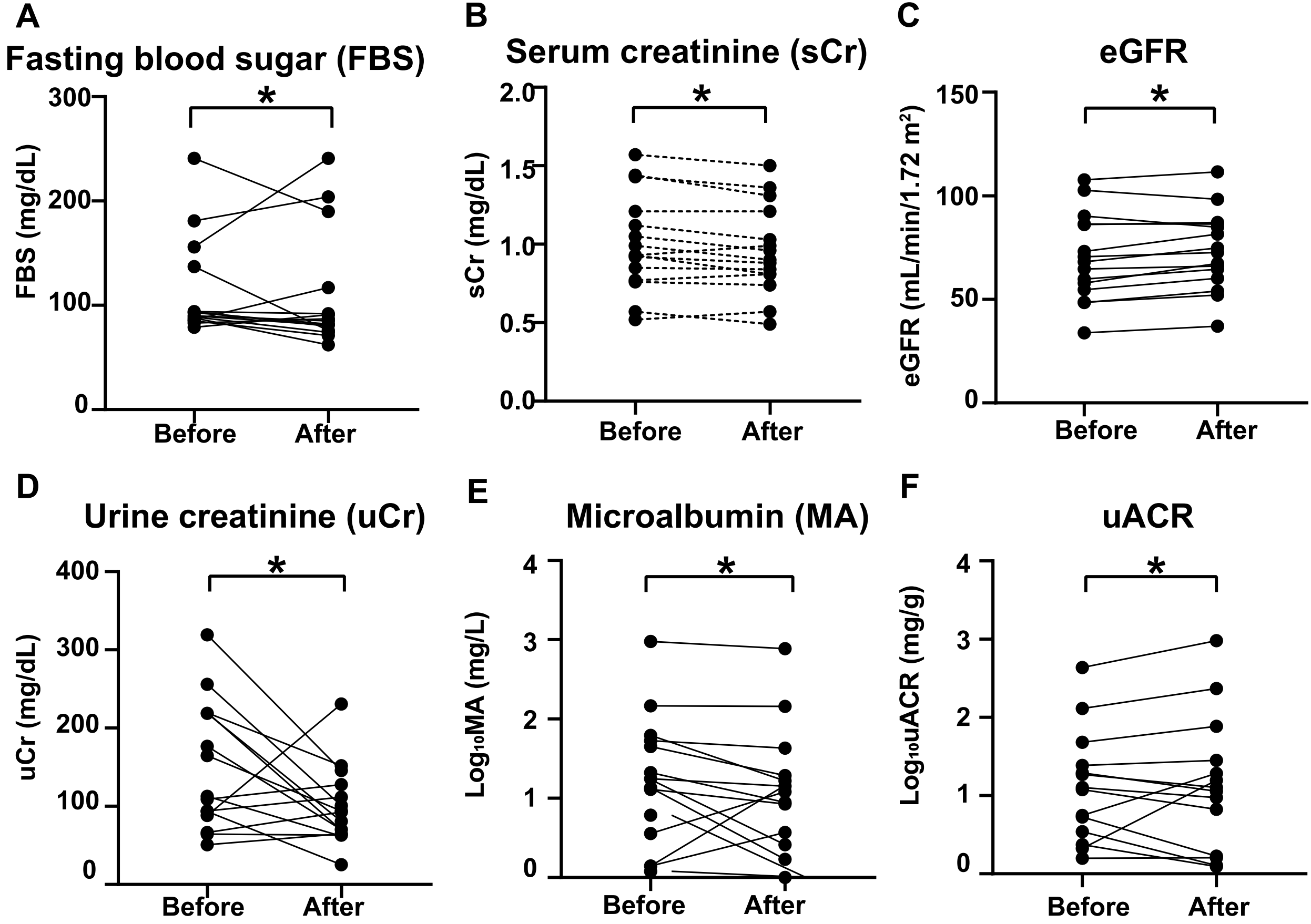

A
Fasting blood sugar (FBS)
FBS (mg/dL)
300
200
100
0
*
Before
After
B
Serum creatinine (sCr)
sCr (mg/dL)
2.0
1.5
1.0
0.5
0.0
*
Before
After
C
eGFR
eGFR (mL/min/1.72 m²)
150
100
50
0
*
Before
After
D
Urine creatinine (uCr)
uCr (mg/dL)
400
300
200
100
0
*
Before
After
E
Microalbumin (MA)
Log₁₀MA (mg/L)
4
3
2
1
0
*
Before
After
F
uACR
Log₁₀uACR (mg/g)
4
3
2
1
0
*
Before
After
