# Supplementary material for: Clearance of Strongyloides stercoralis infection using ivermectin improves kidney function and modulates untargeted urinary metabolomic profile in patients with early-stage chronic kidney disease
Source: PeerJ. 2026 Oct 29;14:e21832. doi: 10.7717/peerj.21832 (PMC13724049; doi:10.7717/peerj.21832)
Supplement: Supplementary file 3 [file peerj-14-21832-s003.pdf]

Clearance of *Strongyloides stercoralis* infection using ivermectin improves kidney function and modulates untargeted urinary metabolomic profile in patients with early-stage chronic kidney disease

Keerapach Tunbenjasiri [1,2], Suppakrit Kongsintaweesuk [1,2], Kornpatchara Chaiyachate [3], Aye Myat Mon [3], Thatsanapong Pongking [2,4], Apinya Jusakul [5], Sirirat Anutrakulchai [2,6], Apisit Chaidee [2,4], Porntip Pinlaor [2,5], Nuttanan Hongsrichan [2,4], Ubon Cha'on [2,7], David Blair [8], Somchai Pinlaor [2,4,*]

[1]Biomedical Sciences Program, Graduate School, Khon Kaen University, Khon Kaen 40002, Thailand

[2]Chronic Kidney Disease Prevention in Northeastern Thailand, Khon Kaen University, Khon Kaen 40002, Thailand

[3]Faculty of Associated Medical Sciences, Khon Kaen University, Khon Kaen 40002, Thailand

[4]Department of Parasitology, Faculty of Medicine, Khon Kaen University, Khon Kaen 40002, Thailand

[5]Centre for Research and Development of Medical Diagnostic Laboratories, Faculty of Associated Medical Sciences, Khon Kaen University, Khon Kaen, Thailand

[6]Department of Medicine, Faculty of Medicine, Khon Kaen University, Khon Kaen 40002, Thailand

[7]Department of Biochemistry, Faculty of Medicine, Khon Kaen University, Khon Kaen 40002, Thailand

[8]College of Science and Engineering, James Cook University, Townsville, Queensland 4811, Australia

* Corresponding Author: Somchai Pinlaor; E-mail: psomec@kku.ac.th

Supplementary Data S1. PCR primer sequences and amplification conditions used for confirmation of clearance of *Strongyloides stercoralis* infection (PDF).

2.5 PCR to confirm *S. stercoralis* infection

Conventional PCR was used to confirm post-treatment stool examination results for *S. stercoralis* infection. Primers described in a previous study were used to amplify a sequence specific to *S. stercoralis* (GenBank accession no. M84229.1). The forward primer was 5′-CCGGACACTATAAGGATTGA-3′, and the reverse was 5′-ACAGACCTGTTATCGCTCTC-3′ (Iamrod et al. 2021). The amplification conditions consisted of an initial denaturation at 94 °C for 5 min; 35 cycles of denaturation at 94 °C for 40 s, annealing at 52.8 °C for 30 s, and extension at 72 °C for 2 min; followed by a final extension at 72 °C for 10 min. PCR products were analyzed by electrophoresis on a 2% agarose gel to confirm amplification and verify the expected amplicon size of 230 bp.

Reference

Iamrod, K. *et al.* Development and Efficacy of Droplet Digital PCR for Detection of Strongyloides stercoralis in Stool. *Am J Trop Med Hyg* **106**, 312-319 (2021).
